# Logging cuts the functional importance of invertebrates in tropical rainforest

**DOI:** 10.1038/ncomms7836

**Published:** 2015-04-13

**Authors:** Robert M. Ewers, Michael J. W. Boyle, Rosalind A. Gleave, Nichola S. Plowman, Suzan Benedick, Henry Bernard, Tom R. Bishop, Effendi Y. Bakhtiar, Vun Khen Chey, Arthur Y. C. Chung, Richard G. Davies, David P. Edwards, Paul Eggleton, Tom M. Fayle, Stephen R. Hardwick, Rahman Homathevi, Roger L. Kitching, Min Sheng Khoo, Sarah H. Luke, Joshua J. March, Reuben Nilus, Marion Pfeifer, Sri V. Rao, Adam C. Sharp, Jake L. Snaddon, Nigel E. Stork, Matthew J. Struebig, Oliver R. Wearn, Kalsum M. Yusah, Edgar C. Turner

**Affiliations:** 1 Department of Life Sciences, Imperial College London, Silwood Park Campus, Buckhurst Road, Ascot SL5 7PY, UK; 2 Faculty of Science, University of South Bohemia, Branisovska 31, Ceske Budejovice CZ-370 05, Czech Republic; 3 Institute of Entomology, Biology Centre of Czech Academy of Sciences, Branisovska 31, Ceske Budejovice CZ-370 05, Czech Republic; 4 Faculty of Sustainable Agriculture, Universiti Malaysia Sabah, Locked Bag No. 3, Sandakan, Sabah 90509, Malaysia; 5 Institute for Tropical Biology and Conservation, Universiti Malaysia Sabah, Jln UMS, Kota Kinabalu, Sabah 88400, Malaysia; 6 School of Environmental Sciences, University of Liverpool, Liverpool L69 3GP, UK; 7 Centre for Invasion Biology, Department of Zoology and Entomology, University of Pretoria, Pretoria 0002, South Africa; 8 Forest Research Centre (Sepilok), Sabah Forestry Department, PO Box 1407, Sandakan, Sabah 90715, Malaysia; 9 School of Biological Sciences, University of East Anglia, Norwich NR4 7TJ, UK; 10 Department of Animal and Plant Sciences, University of Sheffield, Western Bank, Sheffield S10 2TN, UK; 11 Entomology Department, Natural History Museum, Cromwell Road, London SW7 5BD, UK; 12 Environmental Futures Research Institute and Griffith School of the Environment, Griffith University, Nathan, Queensland 4111, Australia; 13 Department of Zoology, University of Cambridge, Downing Street, Cambridge CB2 3EJ, UK; 14 School of Rural, Animal and Environmental Sciences, Nottingham Trent University, Brackenhurst Campus, Southwell, Nottinghamshire NG25 0QF, UK; 15 Centre for Biological Sciences, University of Southampton, Southampton SO17 1BJ, UK; 16 Durrell Institute of Conservation and Ecology, School of Anthropology and Conservation, University of Kent, Canterbury CT2 7NR, UK; 17 Institute of Zoology, Zoological Society of London, Regent's Park, London NW1 4RY, UK

## Abstract

Invertebrates are dominant species in primary tropical rainforests, where their abundance and diversity contributes to the functioning and resilience of these globally important ecosystems. However, more than one-third of tropical forests have been logged, with dramatic impacts on rainforest biodiversity that may disrupt key ecosystem processes. We find that the contribution of invertebrates to three ecosystem processes operating at three trophic levels (litter decomposition, seed predation and removal, and invertebrate predation) is reduced by up to one-half following logging. These changes are associated with decreased abundance of key functional groups of termites, ants, beetles and earthworms, and an increase in the abundance of small mammals, amphibians and insectivorous birds in logged relative to primary forest. Our results suggest that ecosystem processes themselves have considerable resilience to logging, but the consistent decline of invertebrate functional importance is indicative of a human-induced shift in how these ecological processes operate in tropical rainforests.

## Abstract (web summary)

Invertebrates are key components in the ecological functioning of tropical forests. Here, Ewers *et al*. show that, compared to primary forest, logging halves the contribution of invertebrate species to several key ecosystem processes, including litter decomposition.

## Graphical abstract

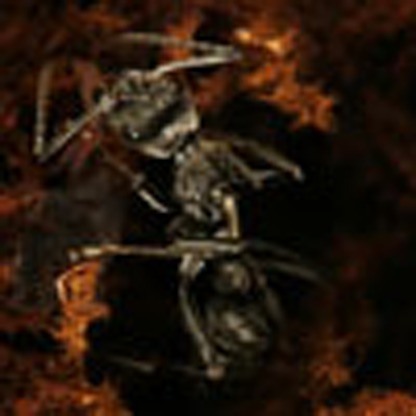

Invertebrates are ‘the little things that run the world'^1^, and nowhere is this more evident than in tropical rainforests. Invertebrates are dominant prey^2^, predators of seeds^3^ and other invertebrates^2^,^4^, herbivores^5^ and pollinators^6^,^7^ in rainforest ecosystems, and are among the most important organisms for breaking down dead organic matter^8^. Tropical rainforests are estimated to host around six million invertebrate species^9^,^10^, and over 18,000 species can be present in a single hectare^11^. This diversity is expected to confer substantial redundancy to ecosystem processes. Differences in environmental sensitivity among functionally similar species give stability to ecosystem processes in the face of environmental change, as the loss of environmentally sensitive species will be compensated for by more robust species^12^. Such functional resistance to disturbance would indicate a resilient ecosystem^13^, and understanding the resilience of natural habitats represents one of the great challenges for predicting the future impacts of human-caused global change on biodiversity^14^.

We conducted a set of experiments to quantify the resistance to intensive logging of three ecosystem processes that operate at three different trophic levels in the tropical rainforests of Borneo. The logged forest had been logged twice^15^, the second time a salvage logging rotation that ended <10 years before data collection, and was conducted under a regime in which any sized tree could be cut. In total, a cumulative amount of 179 m^3^ ha^−1^ timber was removed^16^, placing our sites among the most heavily logged forests in the tropics^17^. Overall, we find that ecosystem processes are remarkably resilient to very heavy logging, but the taxa performing those processes change. Ecosystem processes in primary forest are dominated by the actions of invertebrates such as ants, termites, beetles and earthworms. In logged forest, however, invertebrates are much less dominant and we find the actions of vertebrate taxa, such as birds, amphibians and small mammals, increase in importance. Our results are consistent with those expected in a resilient ecosystem, with that resilience conferred through functional similarity between invertebrates and vertebrates.

## Results

### Resilience of ecosystem processes

**Figure 1:**
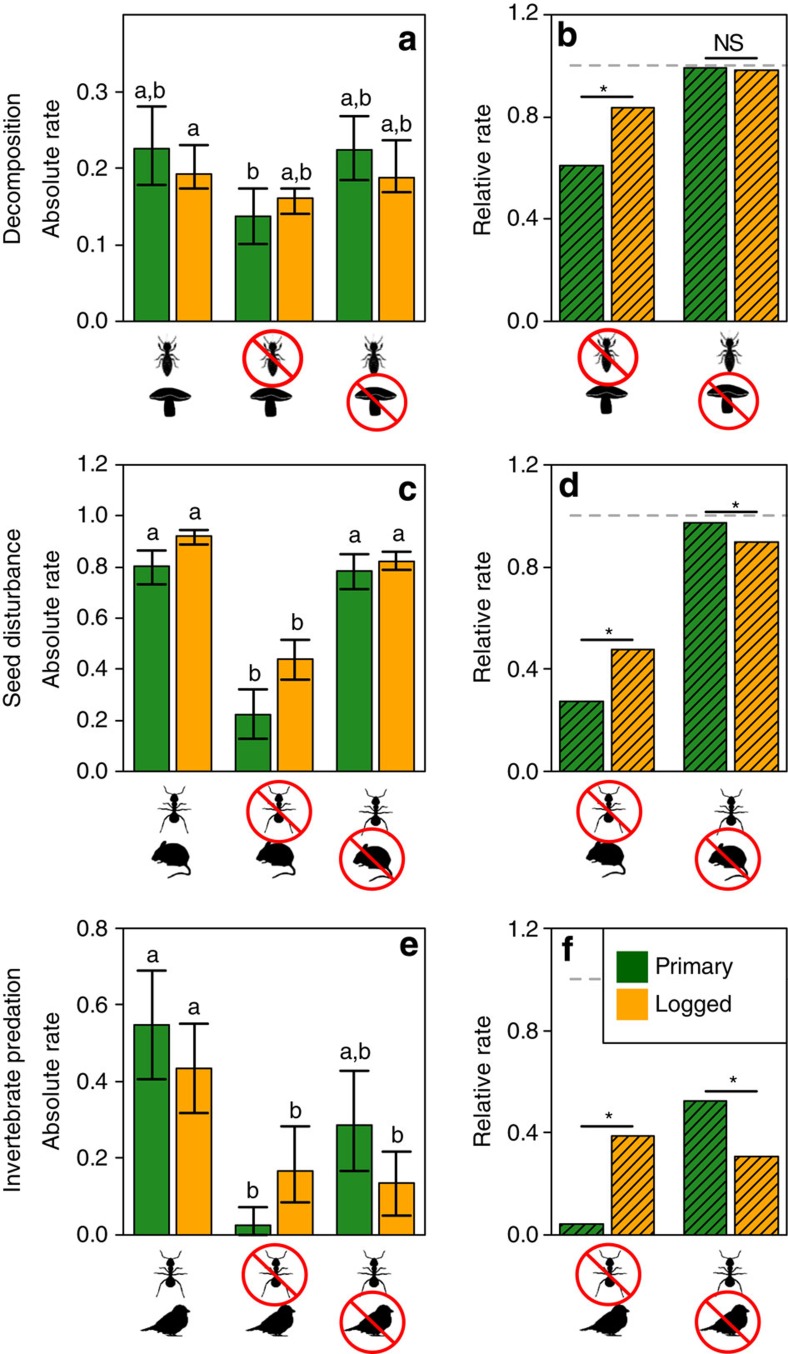
Ecosystem process rates in primary (green) and logged (orange) forest in response to experimental removal of invertebrates, fungi or vertebrates. Ecosystem processes were quantified at three trophic levels represented by (**a**,**b**) leaf litter decomposition rate, (**c**,**d**) seed disturbance, defined as the combined removal and/or predation rate; and (**e**,**f**) invertebrate predation rate. Symbols indicate the taxa that contributed to the rates displayed. Absolute values (mean±95% CI) of the ecosystem process rates are presented in the left-hand column and are measured as (**a**) the slope of a linear regression model relating log_e_-transformed litter mass (g) as a function of log_e_-transformed time (number of days), (**c**) the proportion of experimental seeds removed or predated per day, and (**e**) the proportion of experimental mealworm larvae predated per day. Letters indicate habitat × treatment categories that did not significantly differ from each other (*P*<0.05). There was a significant treatment × habitat interaction for all three ecosystem processes, demonstrating that the role of invertebrates was stronger in primary than logged forests. In the right-hand column (panels **b**,**d**,**f**), values represent the proportional change in ecosystem process rates relative to control sites (calculated from data presented in the left-hand column). Values <1 (dashed line) indicate functions whose rate is reduced following the exclusion of a taxon; smaller values indicate larger reductions in the rate and hence a stronger contribution of that taxon to delivering the ecosystem process. Posthoc significance tests were used to examine the treatment × habitat interaction effects. For example, in panel **b**, the asterisk indicates that the effect on decomposition of excluding invertebrates was significantly (*P*<0.05) larger in primary forest than in logged forest. NS indicates nonsignificant interactions.

We found a 15% decrease in the rate of leaf litter decomposition (likelihood ratio test, *χ*^2^_(1)_=5.90, *P*=0.015, *N*=25) in logged compared with primary forest, a 13% increase in the rate at which seeds were removed and/or predated (likelihood ratio test, *χ*^2^_(1)_=3.98, *P*=0.047, *N*=194), and no difference in invertebrate predation rate (Fig. 1; likelihood ratio test, *χ*^2^_(1)_=1.03, *P*=0.310, *N*=51; Supplementary Table 1). In combination, this indicates considerable resilience of ecosystem processes to the biodiversity changes caused by logging. However, experimental manipulations demonstrated that invertebrates contributed significantly less to delivering all three ecosystem processes in logged relative to primary forest (Fig. 1).

### Invertebrate contributions to ecosystem processes

**Figure 2:**
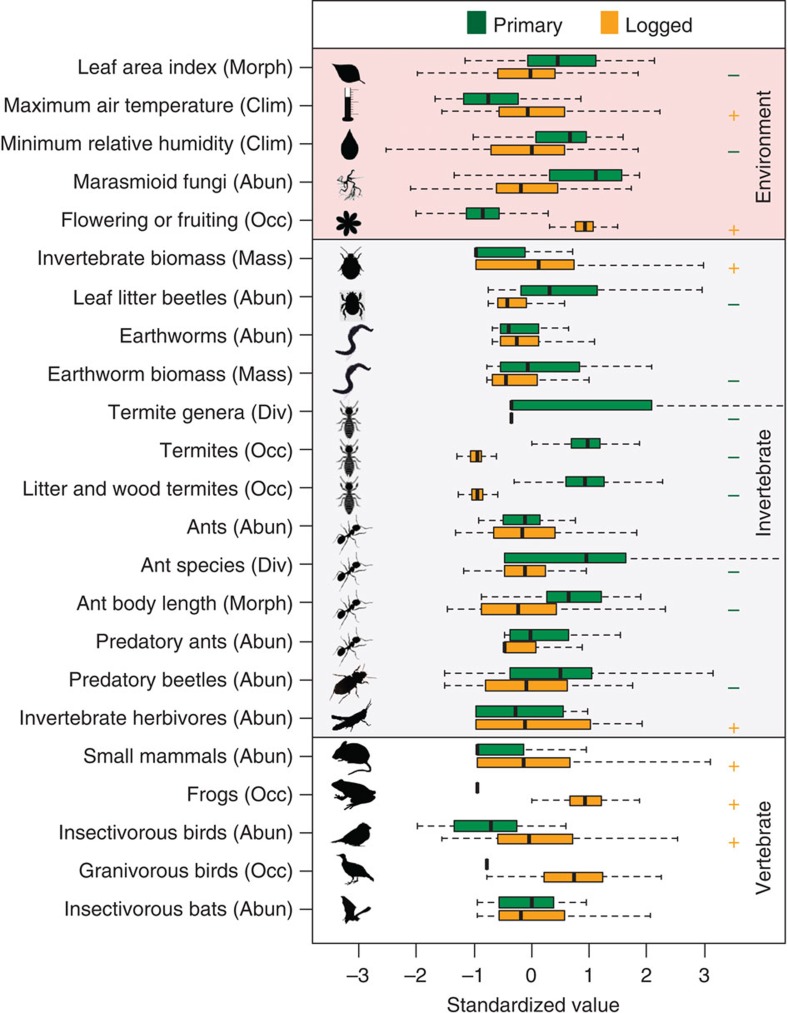
Differences in the physical environment and the functional composition of invertebrate and vertebrate communities between primary and logged tropical rainforest. Response variables fall into the categories of microclimate (Clim), morphology and structure (Morph), biomass (Mass), diversity (Div), occurrence (Occ) and abundance (Abun). Invertebrate groups tend to be more abundant in primary than in logged forest, whereas the reverse is generally true for vertebrate groups. For comparison, all values were standardized to represent standard deviations from the mean. Dark lines represents the median, boxes the first and third quartiles, and whiskers the range. Significant differences (*P*<0.05) from mixed effect models on untransformed data are indicated with a green minus (−) or orange plus (+) sign if the effect of logging was negative or positive.

We quantified the rate of decomposition of *Macaranga* sp. leaves, a common genus of early successional trees in southeast Asian rainforests found in both primary and logged forest. When invertebrates were excluded from leaf litter, decomposition rates were reduced by 39% in primary forest but by just 16% in logged forest (Fig. 1a,b), meaning the invertebrate contribution to decomposition processes in logged forest is less than one-half of that in primary forest (habitat × treatment interaction effect: likelihood ratio test, *χ*^2^_(2)_=41.48, *P*<0.001, *N*=25; Supplementary Table 2). This experiment does not, however, account for potential bias that may arise from differential invertebrate preferences for different litter types in the primary and logged forest habitats. Treating litter with a broad-spectrum fungicide had little effect, reducing decomposition rates by <2% in both habitats, suggesting that bacteria rather than fungi are important in the early phases of leaf decomposition in these forests^18^. Soil bacteria in Borneo are known to be resistant to logging^19^ and have been shown to be more resistant to the logging associated compaction of soil than fungi^20^. At our site there was a reduction, albeit nonsignificant, in the abundance of above-ground litter-trapping marasmioid fungal networks^21^ in logged relative to primary forest (Fig. 2; likelihood ratio test, *χ*^2^_(1)_=3.76, *P*=0.052, *N*=114).

We placed seeds on the forest floor to quantify the rate at which seeds were disturbed (either predated and/or removed) over a 24-h period. Excluding invertebrates from seeds in primary forest reduced the seed disturbance rate by almost three quarters (72%), but their role was smaller in logged forest, where excluding invertebrates reduced the rate of seed disturbance by only one-half (52%; Fig. 1c,d; habitat × treatment interaction effect: likelihood ratio test, *χ*^2^_(2)_=46.19, *P*<0.001, *N*=194; Supplementary Table 2). By contrast, vertebrates, primarily small mammals, accounted for just 3% of seed disturbance in primary forest, but their importance increased significantly in logged forest where they accounted for 10% of all seed disturbance.

We found similar results in predation experiments quantifying the rate at which live mealworms tethered to artificial leaves were predated. Predation rates decreased by 96% in primary forest when invertebrates were excluded (Fig. 1f). In logged forest, however, invertebrates contributed just 62% of all predation (Fig. 1e,f; habitat × treatment interaction effect: likelihood ratio test, *χ*^2^_(2)_=10.77, *P*=0.005, *N*=51; Supplementary Table 2). Excluding vertebrates, such as insectivorous birds and bats and small mammals such as treeshrews that forage in the understorey, also reduced predation of invertebrates in primary forest (48%), but their contribution to predation was significantly higher in logged forest (69%).

### Altered functional compositional of the rainforest fauna

To understand why the relative importance of invertebrates versus vertebrates changes after logging, we conducted a comprehensive assessment of the functional diversity of the rainforest fauna in primary and logged forest. We dug soil pits to obtain data on the occurrence patterns of 27 genera of termite (*N*=429 individuals) and 416 earthworms, and made field observations on the abundance of 192 species of foraging ant (*N*=4,173). We used canopy fogging to collect invertebrate herbivores (*N*=1,492), and modified flight intercept/pitfall traps to quantify invertebrate biomass, the abundance of leaf litter beetles (*N*=1,820) and occupancy of leaf litter frogs (collected as by-catch; *N*=33). Harp traps were used to capture 26 species of bat (*N*=687), point counts to identify 115 species of understorey bird (*N*=1,972) and live-capture traps to collect 26 species of small mammals (*N*=1,897).

The reduced role of invertebrates in decomposition processes is probably due to declines in the abundance of key invertebrate decomposers in logged forest (Supplementary Table 3). The abundance of leaf litter beetles (Fig. 2; likelihood ratio test, *χ*^2^_(1)_=10.87, *P*<0.001, *N*=198), the occurrence of termites (Fig. 2; likelihood ratio test, *χ*^2^_(1)_=4.14, *P*=0.042, *N*=41), including those belonging to the functional group associated with eating dead wood, grass or leaf litter (Fig. 2; likelihood ratio test, *χ*^2^_(1)_=5.87, *P*=0.015, *N*=41), and the genus-level diversity of termites (Fig. 2; likelihood ratio test, χ^2^_(1)_=4.51, *P*=0.034, *N*=41) were all reduced by two-thirds in logged relative to primary forest. In addition, the biomass of earthworms in logged forest was almost half of that in primary forest (Fig. 2; likelihood ratio test, *χ*^2^_(1)_=5.41, *P*=0.020, *N*=27), although their abundance remained constant (Fig. 2; likelihood ratio test, *χ*^2^_(1)_=0.44, *P*=0.506, *N*=27), probably because individual worms in primary forest have larger body sizes. The most likely explanation for reductions in the abundance or biomass of these key functional groups is altered microclimate (Supplementary Table 4). Logging opens gaps in the rainforest canopy and logged forests therefore have a lower leaf area index (Fig. 2; likelihood ratio test, *χ*^2^_(1)_=5.51, *P*=0.019, *N*=98) resulting in higher daily maximum air temperature^22^ (Fig. 2; likelihood ratio test, *χ*^2^_(1)_=5.82, *P*=0.016, *N*=161) and lower minimum humidity (Fig. 2; likelihood ratio test, *χ*^2^_(1)_=6.79, *P*=0.009, *N*=161) than primary forests. Soft-bodied invertebrates such as earthworms and termites are particularly sensitive to desiccation^23^, and their decline in logged forests is consistent with the altered microclimate patterns we observed.

Invertebrates, including leaf litter beetles and ants, are assumed to be dominant predator and seed disturbance agents in tropical rainforests^4^, and we found substantial changes in the abundance and functional composition of these communities in logged forest (Supplementary Table 5). There was no difference in the total abundance of ants foraging in the leaf litter in logged relative to primary forest (Fig. 2; likelihood ratio test, *χ*^2^_(1)_=0.01, *P*=0.912, *N*=210), but there were 25% fewer ant species (Fig. 2; likelihood ratio test, *χ*^2^_(1)_=6.74, *P*=0.009, *N*=210), the average body size of foraging ant species was one-third smaller (Fig. 2; likelihood ratio test, *χ*^2^_(1)_=4.73, *P*=0.030, *N*=210), and the abundance of predatory beetles (Fig. 2; likelihood ratio test, *χ*^2^_(1)_=5.04, *P*=0.025, *N*=198) and predatory ants (Fig. 2; likelihood ratio test, *χ*^2^_(1)_=3.41, *P*=0.065, *N*=210) in the leaf litter were halved, although this latter difference was not statistically significant. Large-bodied, carnivorous ants are known to be particularly sensitive to land use change in Borneo^24^, a pattern that may in part arise because of the altered structure of the forest canopy and an increase in generalist, visual vertebrate predators.

Concurrent with the changes to the functional composition of invertebrate communities, the abundance of key vertebrate functional groups tended to increase in logged relative to primary forest (Supplementary Table 6). Capture rates of small mammals at ground level were more than three times higher in logged forest than in primary forest (Fig. 2; likelihood ratio test, *χ*^2^_(1)_=6.47, *P*=0.011, *N*=1,248). Frogs were much more likely to be present on the ground (Fig. 2; likelihood ratio test, *χ*^2^_(1)_=7.62, *P*=0.006), and there was a significantly higher abundance of insectivorous birds (Fig. 2; likelihood ratio test, *χ*^2^_(1)_=10.07, *P*=0.002, *N*=114), in logged than in primary forest. It is unlikely that trophic release explains the increased abundance of small mammals because there is little evidence of trophic release in the region^25^ and the logged forest we worked in has a high abundance of top carnivores^26^. Rather, these vertebrate groups probably increase in abundance because of increased resource availability: vegetation plots were twice as likely to contain trees that were fruiting or flowering (Fig. 2; likelihood ratio test, *χ*^2^_(1)_=4.48, *P*=0.034, *N*=112), and the total invertebrate biomass was doubled (Fig. 2; likelihood ratio test, *χ*^2^_(1)_=11.59, *P*<0.001, *N*=198), in logged relative to primary forest. The increased invertebrate biomass is predominantly comprised of large herbivorous invertebrates (Fig. 2; Supplementary Table 5; likelihood ratio test, *χ*^2^_(1)_=3.94, *P*=0.047, *N*=107). These herbivores do not contribute directly to the ecosystem functions we examined, suggesting that herbivory may be one function that runs counter to the general trend for invertebrates to decline in importance in logged forest. We did not find a definitive effect of logging on the occurrence of granivorous birds, which were rarely recorded at this study site (Fig. 2; likelihood ratio test, *χ*^2^_(1)_=1.53, *P*=0.306, *N*=114), or on the abundance of insectivorous bats^16^ (Fig. 2; likelihood ratio test, *χ*^2^_(1)_=0.53, *P*=0.469). Together, the reduced abundance of large and predatory ants and beetles, combined with the increased abundance of a suite of generalist vertebrates, help explain why the contribution of invertebrates to seed disturbance and predation rates in logged forests decreased while that of vertebrates increased.

## Discussion

Our data suggest that the ecosystem processes operating in tropical forests remain considerably resistant to the human disturbance of high-intensity logging. This occurs despite large changes in the abundance, diversity and functional composition of termites, ants, beetles and earthworms, the key invertebrate groups primarily responsible for those functions in primary forest. The fact that taxa other than invertebrates are able to perform the same ecosystem processes probably confers the resilience to human disturbance. For the decomposition processes, this resilience may potentially arise because of increased physical fracturing of litter under the harsher microclimatic conditions in logged forest or possibly through a density compensation effect^12^ of bacteria or other decomposer micro-invertebrates. For the seed disturbance and invertebrate predation processes, our data indicate the resilience is partially conferred through a density compensation effect of vertebrates. However, the susceptibility of vertebrates to a range of anthropogenic pressures has seen their threat status steadily rise over the last two decades^27^, meaning an increased reliance on vertebrates to deliver ecosystem processes in logged forests may leave these systems vulnerable to future change.

Primary forests remain irreplaceable for conserving tropical biodiversity^28^, but the conservation value of logged forests should not be ignored. Depending on the intensity of selective logging^17^ and the timber-harvesting method used^29^, logged forests can retain high biodiversity value in southeast Asia and elsewhere^28^, supporting many of the tree^30^, bird^31^, bat^16^, cat^26^ and invertebrate^31^ species found in primary forests and justifying decisions to protect large areas of logged forest^32^. But forests are more than the sum of their component species. They are complicated networks of species that interact with each other and with the environment to perform ecological functions such as nutrient cycling, the regulation of water regimes and, ultimately, the modulation of local and global climates. Our data show that ecological processes associated with primary forest are retained in logged forest, but that the role of the world's most diverse taxon in performing those processes is greatly diminished. Invertebrates remain an important actor in logged forests, but human actions are deposing them from their dominant role in rainforest ecosystems.

## Methods

### Study site and design

Data were collected at the Stability of Altered Forest Ecosystems Project in Sabah, Malaysia^15^. Logged forests had been through one round of selective logging (removing 113 m^3^ ha^−1^) in the 1970s and a second round of salvage logging^16^ (removing 66 m^3^ ha^−1^) that occurred between 2000 and 2008. Data collection took place between 2010 and 2012, ∼5–10 years after logging ended.

All data collection had a nested structure with sample sites clustered at up to four spatial scales based on a fractal sampling pattern^15^,^33^ (Supplementary Fig. 1). At the finest scale, sites were separated by 10^1.75^ m (first order), with those clusters of sites separated by 10^2.25^ m (second order) and again at 10^2.75^ m (third order). All third-order sites were nested within blocks separated by >1 km and the average area of a block was 71 ha. There are 17 sampling blocks at the Stability of Altered Forest Ecosystems Project, which vary in the level of historical disturbance^15^. For these analyses, we used data collected from eight blocks: two blocks located in unmodified primary forest (blocks OG1 and OG2) and six blocks located in salvage logged forest (blocks A, B, C, D, E and F). We used larger sample sizes in the logged forest because data on leaf area index, an index of forest structure, demonstrated the habitat there was more variable than in the primary forest, with higher variance (0.51 versus 0.39) and a larger range (0.83–5.56 versus 3.12–5.48) of values. Not all datasets were collected at all points, so sample sizes differ among the datasets used in analyses.

### Ecosystem process experiments

Litter decomposition: naturally senesced whole leaves of *Macaranga* sp. were collected within 24 h of falling. We used a morphospecies (*M*. cf. *pearsonii*) that is a common early successional tree species, is highly abundant within logged forest and also common along riverine margins and in treefall gaps within primary forest. This standardization of the litter does, however, mean our experiment was unable to control for any potential biases in invertebrate preference behaviour among the two habitats. Leaves were cut into roughly 2 cm^2^ pieces, discarding large veins and petioles, and dried to constant weight. Litter pieces were separated into 4-g units and placed in nylon bags with a 1-mm mesh. To allow invertebrates access to the litter, five 1-cm^2^ perforations were made on each side of the bag. Fungi were excluded by treating the filled litter bags with broad-spectrum fungicide containing 40% chlorothalonil^34^. Data were collected at second-order sites with nine primary forest (block OG2) and 16 logged forest (block E) sites. At each site we placed nine litter bags; three each for the control, invertebrate exclosure and fungal exclosure treatments. One bag from each treatment was collected after 14, 27 and 40 days (±2 days), respectively, and on each day, five or six additional bags were carried to and from the field to calculate the amount of litter mass lost through handling. Although a short period, this was long enough to detect invertebrate impacts on decomposition that are consistent with results reported elsewhere in the tropics^34^,^35^,^36^. Collected bags were dried to constant weight. Decomposition rate for each site × treatment combination was quantified as the slope of linear regression modelling litter mass as a function of log_e_-transformed number of days in the field, using handling loss as an offset in the model.

Seed disturbance: to compare seed disturbance rates between vertebrates and invertebrates, we required an intermediate seed size and type that was attractive to both taxa. We conducted pilot trials that indicated large seeds such as peanuts were not disturbed by invertebrates, and small seeds such as sesame were ignored by vertebrates. We found that pumpkin seeds were disturbed by both vertebrates and invertebrates, and thus were chosen for the experiments.

Data were collected at first-order sites with 48 primary forest (block OG2) and 146 logged forest (blocks D and F) sites. At each site we placed 20 pumpkin seeds on each of three standardized brown plastic ‘leaves' (80 × 120 mm) left sitting on the forest floor. One of the three leaves had ground-moving invertebrates excluded by surrounding the leaf with a wooden frame coated with insect-trapping glue, a second had vertebrate predators excluded by placing the artificial leaf inside a 30 × 30 × 30 cm wire cage with a 1-cm mesh, and the third was left as a control. We defined the seed disturbance rate as the proportion of seeds that were either removed from the leaf, or partially eaten but left in place on the leaf, over a 24-h period.

Invertebrate predation: data were collected at first-order sites with 21 primary forest (block OG2) and 30 logged forest (block E) sites. At each site we attached one live larval mealworm (*Tenebrio* sp.) to each of three standardized green plastic ‘leaves' (80 × 120 mm) using fine cotton thread and clear tape. Enough cotton thread was provided to allow the mealworm to crawl around the leaf, and artificial leaves were attached to saplings 1.5 m above ground. One of the three leaves had ground-moving invertebrate predators excluded by applying insect-trapping glue to the sapling stem 20 cm above and below the artificial leaf, a second had vertebrate predators excluded by placing the artificial leaf inside a 30 × 30 × 30 cm wire cage with a 1-cm mesh, and the third was left as a control. Predation of mealworms was recorded over a 24-h period.

### Functional composition of the rainforest fauna

Invertebrate collections: leaf litter beetles were collected at 54 first-order sites in primary forest (blocks OG1 and OG2) and 144 first-order sites in logged forest (blocks A, C and E) over 3 days during the wet season. We used modified flight intercept traps dug into the ground to simultaneously act as a pitfall trap (23 cm diameter × 60 cm high). Traps were part-filled with 70% ethanol to act as a killing agent. Traps collected frogs as by-catch that allowed us to analyse the presence or absence of frogs in relation to habitat. The total invertebrate biomass of samples was estimated by placing all invertebrates with body length >5 mm on blotting paper and weighing the blotted invertebrates. We excluded small invertebrates despite their high abundance because their small body size means they are likely to contribute less to total community biomass^37^,^38^, and also to community-level energy fluxes^37^,^39^, than the less abundant, but larger, organisms. We calibrated sample-level wet weight biomass of invertebrates with sample-level dry weight biomass using 42 samples that were first wet weighed and then oven dried to constant weight. Wet weight was strongly and linearly correlated with dry weight (linear regression through the origin, *F*_1,41_=1,578, *P*<0.001, *R*^2^=0.97, slope=0.217±0.005 s.e.). Canopy invertebrates were collected by fogging at 12 second-order sites in primary forest (block OG2) and 95 second-order sites in logged forest (blocks A, B, C, D, E and F). At each site, four trays of 1-m diameter were laid out with collecting pots filled with 95% ethanol attached. Fog formulation was synthetic pyrethrum insecticide (active compound: alphacypermethrin with synergist 2.27 %) and diluted in diesel by a ratio of 15:1. Fogging activity started at 07:00 and lasted for 4 min at each site. Arthropods were collected after a 2-h period and identified to order.

Termites: we hand-collected termites from 16 soil pits (12 cm diameter × 10 cm deep) at each of nine second-order primary sites (block OG2) and 32 second-order logged forest sites (blocks C and F), and were identified to genus^40^. Earthworms were collected from four soil monoliths per site (three monoliths of 50 × 50 cm wide × 30 cm deep and a fourth, smaller monolith of 25 × 25 cm wide × 30 cm deep) at each of 9 second-order primary sites (block OG2) and 18 second-order logged forest sites (blocks B and F). Foraging ant abundance and species richness was quantified at 18 first-order primary forest and 192 first-order logged forest sites by counting and identifying the number of ants entering a 12 × 14 cm plastic card, laid flat in the leaf litter and baited with 30 compressed dried earthworm pellets, over a 40-min period. All observations were conducted between 10:00 and 15:00.

Invertebrate functional traits: beetles were identified to family and families were classified according to whether they contained predominantly predatory species or not^41^. All foraging ants were identified to genus, split into morphospecies and assigned species names where possible. Termites were also identified to genus and grouped according to feeding position along a four-step humification gradient, with the second group representing those that feed on grass, dead wood and leaf litter^42^. We measured Weber's length, a common proxy for body size in ants, for each of the 192 species of foraging ant, averaging measurements from between one and five minor workers per species. Ant genera were also classified according to whether they belonged to the specialist predator functional group or not^43^. We used the abundance-weighted mean body size (log_10_-transformed) of all ants, and the total abundance of specialist predators, visiting each site as the response variable in analyses. Arthropods from canopy fogging samples belonging to the Orthoptera, Phasmida, Homoptera and Heteroptera were classified as herbivores.

Small mammals: we trapped small mammals within 1.75 ha grids overlaying the nested sampling design used in other data collection. Each grid consisted of a 4 × 12 rectangular arrangement of points separated by 23 m. Two locally made small mammal traps (280 × 140 × 140 mm), baited with oil palm fruit, were placed at or near ground level (≤1,500 mm) within 10 m of each grid point, making 96 traps in total per grid. For this analysis, we included captures only from those traps that lay within 30 m of a first-order sampling point, including 265 traps in primary forest (at 27 first-order primary sites in blocks OG1 and OG2) and 983 traps in logged forest (at 103 first-order sites in blocks D, E and F). Trapping sessions ran for seven consecutive days and we used the capture rate of all species combined (captures per seven days) in the analyses.

Bats: four-bank harp traps were set across trails and logging skids at nine second-order sites in primary forest (block OG2) and 81 second-order sites in logged forest (blocks A, B, C, D, E and F) to target insectivorous bats foraging in the forest understory^22^. Up to seven traps were set each night, 50 to 150-m apart, and moved to a new position the following day. All bats were marked and released at the capture point.

Birds: we sampled birds using 15-minute point counts of 50 m radius at 18 second-order primary forest sites (blocks OG1 and OG2) and 96 second-order logged forest sites (blocks A, B, C, D, E and F). Birds were identified by a single experienced observer (David P. Edwards) and any unknown vocalizations were recorded using a Sennheiser ME-66 directional microphone and Edirol R09-HR digital recorder, and subsequently identified against reference collections available from http://www.xeno-canto.org/. Swifts (Apodidae) and swallows (Hiurundae) were not recorded because they are difficult to observe in closed-canopy forest. We recorded the combined abundance of all bird species classified as belonging to insectivore (including both obligate and generalists) and granivore guilds^44^.

### Environmental variables

Leaf area index was calculated from 13 hemispherical photographs per plot at 18 second-order primary forest sites (blocks OG1 and OG2) and 80 second-order logged forest sites (blocks A, B, C, D, E and F), and processed following the methods of Pfeifer *et al*.^45^. The presence of flowers or fruits in 25 × 25 m vegetation plots was recorded at 18 second-order primary forest sites (blocks OG1 and OG2) and 96 second-order logged forest sites (blocks A, B, C, D, E and F). Within each vegetation plot we counted the number of clumps of the litter-trapping fungi belonging to the genus *Marasmius* spp., which forms abundant and easily recognizable networks of brown rhizomes that trap leaf litter above the ground^21^. We placed iButton DS1923-F5 dataloggers (Dallas Semiconductor) to record air temperature and relative humidity 1-m above ground every 3 h, from which we determined the average maximum daily air temperature and minimum daily humidity in the dry season (March and April)^46^, when the forest experiences the most extreme microclimatic conditions, at 34 first-order primary forest sites (blocks OG1 and OG2) and 127 first-order logged forest sites (blocks A, B, C, D, E and F).

### Statistical analyses

We fitted generalized linear mixed effect models to the data with random effects reflecting the nested structure of data collection (multiple observations within first-order sites within second-order sites within third-order sites within blocks). Error distributions for the models were selected according to the nature of the response variables and are recorded in Supplementary Tables 1–6. Count data, which was used for all estimates of animal abundance and species richness, was modelled with a Poisson error distribution. Presence–absence and occurrence data, along with binary response variables such as invertebrate predation and seed disturbance, were modelled with a binomial error distribution. All other variables were modelled using Gaussian errors, with response variables log_10_-transformed where this improved normality. All models were fitted using the lme4 package^47^ of the R statistical computing environment^48^. We used likelihood ratio tests to determine parameter significance by comparing models with habitat (primary versus logged forest) to a null model with no predictor. The proportion of variance explained by fixed effects 
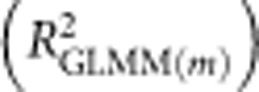
 was calculated and is reported in Supplementary Tables 1–6.

Posthoc significance tests using the glht function in the multcomp package^49^ were used to compare treatment effects and their interactions. We determined the proportion of variance explained by fixed effects and by the model as a whole including variance explained by the random effects^50^. Residuals of all models were tested for spatial autocorrelation to estimate the spatial dependence of model residuals as a continuous function of distance^51^. In no case did we detect significant spatial autocorrelation (Supplementary Tables 1–6) so this is not discussed further. Full results of the partitioning of variance explained and tests of spatial autocorrelation are presented in Supplementary Tables 1–6.

## Author contributions

R.M.E. designed the study, conducted all analyses and wrote the first draft of the manuscript. R.M.E., M.J.W.B., R.A.G., N.S.P., T.R.B., R.G.D., D.P.E., P.E., T.M.F., S.R.H., R.L.K., K.M.S., S.H.L., J.J.M., M.P., S.V.R., A.C.S., N.E.S., M.J.S., O.R.W., K.M.Y. and E.C.T. contributed the data. All authors commented on manuscript drafts.

## Additional information

**How to cite this article:** Ewers, R. M. *et al*. Logging cuts the functional importance of invertebrates in tropical rainforest. *Nat. Commun*. 6:6836 doi: 10.1038/ncomms7836 (2015).

## Supplementary Material

Supplementary Information Supplementary Figure 1, Supplementary Tables 1-6 and Supplementary Reference
