# Supplementary material for: Logging cuts the functional importance of invertebrates in tropical rainforest
Source: Nat Commun. 2015 Apr 13;6:6836. doi: 10.1038/ncomms7836 (PMC4403313; doi:10.1038/ncomms7836)
Supplement: Supplementary Information — Supplementary Figure 1, Supplementary Tables 1-6 and Supplementary Reference [file ncomms7836-s1.pdf]

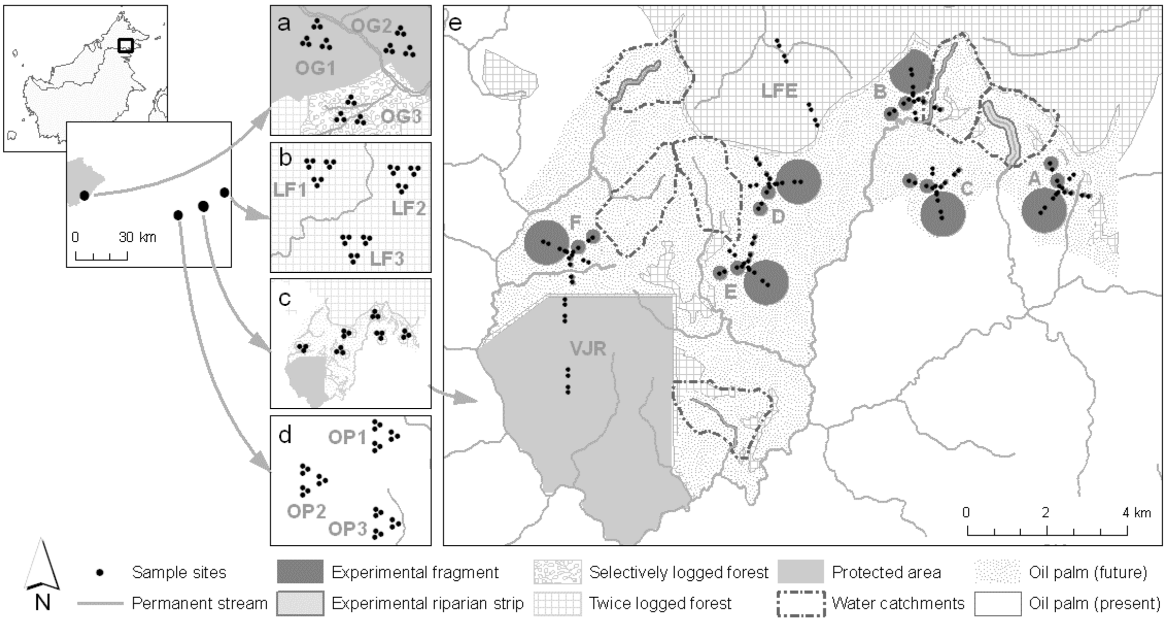

Supplementary Fig. 1. Design of the sampling scheme used for collecting data at the SAFE Project in Malaysian Borneo. Here, we used data collected from sample blocks in primary forest (Blocks OG1 and OG2) and from twice-logged forest that was continuous at the time of data collection, but will become isolated forest fragments in the future (Blocks A-F). Displayed sample sites are second-order points. Figure reproduced from Ewers et al.[1]

Supplementary Table 1. Mixed effects modelling of the effects of logging on three ecosystem process rates: (a) leaf litter decomposition rate, (b) seed disturbance rate and (c) invertebrate predation rate.

Ecosystem function:	(a) Decomposition rate		(b) Seed disturbance rate		(c) Invertebrate predation rate	
	Linear mixed model		Binomial mixed model (logit link)		Binomial mixed model (logit link)	
Model name	Null model	Full model	Null model	Full model	Null model	Full model
Fixed effects	*b* [95% CI]	*b* [95% CI]	*b* [95% CI]	*b* [95% CI]	*b* [95% CI]	*b* [95% CI]
Intercept	-0.2 [-0.22, -0.19]	-0.19 [-0.21, -0.18]	-2.88 [-3.96, -1.79]	-1.54 [-2.87, -0.22]	-0.08 [-0.55, 0.38]	0.2 [-0.51, 0.91]
Habitat (logged)	-	-0.03 [-0.06, -0.01]	-	-1.87 [-3.38, -0.35]	-	-0.48 [-1.42, 0.45]
Random effects	VC	VC	VC	VC	VC	VC
1st order	-	-	-	-	0.0000	0.0157
2nd order	-	-	1.8414	1.9500	0.0259	0.2041
3rd order	0.0004	0.0002	0.9049	0.7033	0.0000	0.0000
Block	-	-	0.4997	0.0000	-	-
Residuals (additive dispersion)	0.0006	0.0006	-	-	-	-
Fixed factors	-	0.0003	-	0.6031	-	0.0575
$PCV_{[1st\ order]}$		-		-		-28067878.53%
$PCV_{[2nd\ order]}$		-		-5.90%		-688.34%
$PCV_{[3rd\ order]}$		53.58%		22.28%		99.40%
$PCV_{[Block]}$		-		100.00%		-
$PCV_{[Residuals]}$		2.59%		-		-
$R^2_{GLMM(m)}$		27.19%		9.21%		1.61%
$R^2_{GLMM(c)}$		44.57%		49.74%		7.77%
AIC	-92.7	-89.2	1554.9	1553.0	148.4	149.4
BIC	-89.0	-84.4	1568.9	1570.4	158.9	162.5
Spatial autocorrelation (residuals)						
x-intercept		-1.22 [-68.21, 1.16]		-0.49 [-4.93, 0.19]		-40.48 [-44.72, 0.57]
y-intercept		-0.14 [-0.98, 0.34]		-0.08 [-0.2, 0]		-0.01 [-0.2, 0.22]

For full models, the intercept represents the primary forest control. CI = Confidence interval; PCV = Proportion change in variance; NA = Not applicable/available; AIC = Akaike Information Criterion; BIC = Bayesian Information Criterion; VC = Variance components

Supplementary Table 2. Mixed effects modelling testing for an interaction effect between logging and experimental treatment on three ecosystem process rates: (a) leaf litter decomposition rate, (b) seed disturbance rate and (c) invertebrate predation rate.

Ecosystem function:	(a) Decomposition rate<br>Linear mixed model		(b) Seed disturbance rate<br>Binomial mixed model (logit link)		(c) Invertebrate predation rate<br>Binomial mixed model (logit link)	
Model name	Null model	Full model	Null model	Full model	Null model	Full model
Fixed effects	*b* [95% CI]	*b* [95% CI]	*b* [95% CI]	*b* [95% CI]	*b* [95% CI]	*b* [95% CI]
Intercept	-0.21 [-0.23, -0.19]	-0.23 [-0.25, -0.21]	-1.74 [-2.74, -0.74]	-1.5 [-2.5, -0.5]	0.08 [-0.52, 0.67]	0.21 [-0.47, 0.9]
Treatment (no inverts)	0.05 [0.04, 0.07]	0.09 [0.08, 0.1]	2.91 [2.78, 3.03]	2.81 [2.58, 3.03]	-2.21 [-2.98, -1.44]	-4.13 [-6.35, -1.91]
Treatment (no other)	0 [-0.01, 0.02]	0 [-0.01, 0.02]	0.7 [0.58, 0.82]	0.13 [-0.08, 0.34]	-1.47 [-2.12, -0.83]	-1.23 [-2.15, -0.31]
Habitat (logged)	0.02 [-0.01, 0.04]	0.03 [0.01, 0.06]	-0.79 [-1.95, 0.37]	-1.16 [-2.33, 0.01]	-0.28 [-0.97, 0.41]	-0.51 [-1.41, 0.39]
Treatment (no inverts) : Habitat (logged)	-	-0.06 [-0.08, -0.04]	-	0.19 [-0.08, 0.46]	-	2.65 [0.26, 5.03]
Treatment (no other : Habitat (logged)	-	0 [-0.02, 0.02]	-	0.84 [0.57, 1.1]	-	-0.54 [-1.86, 0.78]
Random effects	VC	VC	VC	VC	VC	VC
1st order	-	-	-	-	0.48	0.05
2nd order	0.00013	0.00023	0.74	0.74	0.00	0.00
3rd order	0.00018	0.00018	0.20	0.20	0.00	0.00
Block	-	-	0.11	0.11	-	-
Residuals (additive dispersion)	0.00049	0.00021	-	-	-	-
Fixed factors	-	0.00080	-	1.63	-	1.60
$PCV_{[1st\ order]}$		-		-		73.28%
$PCV_{[2nd\ order]}$		NA	NA	-59.28%	NA	NA
$PCV_{[3rd\ order]}$		-5.77%	NA	-20.48%	NA	NA
$PCV_{[Block]}$		-	NA	37.58%	-	-
$PCV_{[Residuals]}$		82.81%	NA	-	-	-
$R^2_{GLMM(m)}$	44.39%	56.49%	26.48%	27.37%	18.74%	29.66%
$R^2_{GLMM(c)}$	65.98%	85.25%	44.30%	44.85%	29.02%	39.13%
AIC	-278.97	-299.06	6927.9	7608.63	321.96	315.19
BIC	-263.04	-278.57	6979.56	7649.33	348.03	348.70
Spatial autocorrelation (residuals)						
x-intercept		-0.5 [-67.64, 3]		-0.27 [-1.98, -0.14]		-0.06 [-0.19, 0.28]
y-intercept		-0.21 [-0.21, 0.14]		-0.02 [-0.15, -0.05]		-0.03 [-0.14, 0.13]

For full models, the intercept represents the experimental control in primary forest habitat. CI = Confidence interval; PCV = Proportion change in variance; NA = Not applicable/available; AIC = Akaike Information Criterion; BIC = Bayesian Information Criterion; VC = Variance components

Supplementary Table 3. Mixed effects modelling testing for the effects of logging on seven aspects of invertebrate community composition: (a) beetle abundance, (b) termite occupancy, (c) litter and wood termite occupancy, (d) termite genus richness, (e) earthworm abundance, (f) earthworm biomass and (g) total invertebrate biomass.

Response:	(a) Beetle abundance<br>Poisson mixed model (log link)		(b) Termite occupancy<br>Binomial mixed model (logit link)		(c) Litter and wood termite occupancy<br>Binomial mixed model (logit link)	
Model name	Null model	Full model	Null model	Full model	Null model	Full model
Fixed effects	*b* [95% CI]	*b* [95% CI]	*b* [95% CI]	*b* [95% CI]	*b* [95% CI]	*b* [95% CI]
Intercept	2.04 [1.53, 2.56]	2.74 [2.43, 3.05]	-2.16 [-3.13, -1.19]	-1.07 [-2.01, -0.14]	-2.74 [-3.54, -1.93]	-1.79 [-2.49, -1.1]
Habitat (logged)	-	-1.14 [-1.52, -0.75]	-	-1.56 [-2.66, -0.45]	-	-1.34 [-2.16, -0.52]
Random effects	VC	VC	VC	VC	VC	VC
1st order	-	-	-	-	-	-
2nd order	0.031	0.302	0.596	0.603	0.495	0.050
3rd order	0.000	0.000	0.175	0.169	0.046	0.000
Block	0.316	0.018	0.576	0.060	0.338	0.000
Residuals (additive dispersion)	-	-	-	-	-	-
Fixed factors	-	0.258	-	0.416	-	0.416
$PCV_{[1st\ order]}$		-		-		-
$PCV_{[2nd\ order]}$		-879.26%		-1.21%		89.84%
$PCV_{[3rd\ order]}$		NA		3.32%		100.00%
$PCV_{[Block]}$		94.35%		89.63%		100.00%
$PCV_{[Residuals]}$		-		-		-
$R^2_{GLMM(m)}$		37.06%		9.17%		7.56%
$R^2_{GLMM(c)}$		NA		27.49%		19.82%
AIC	1664.8	1655.9	478.7	476.6	344.2	340.4
BIC	1677.9	1672.3	496.7	499.0	362.2	362.8
Spatial autocorrelation (residuals)						
x-intercept		-0.75 [-4.7, -0.14]		-0.43 [-3.25, 1.57]		-0.42 [-2.76, 1]
y-intercept		-0.09 [-0.16, -0.01]		-0.02 [-0.06, 0.06]		-0.02 [-0.06, 0.04]

Supplementary Table 3 cont.

Response:	(d) Termite genus richness Poisson mixed model (log link)		(e) Earthworm abundance Poisson mixed model (log link)		(f) Earthworm biomass Linear mixed model	
Model name	Null model	Full model	Null model	Full model	Null model	Full model
Fixed effects	b [95% CI]	b [95% CI]	b [95% CI]	b [95% CI]	b [95% CI]	b [95% CI]
Intercept	-2.22 [-3.06, -1.38]	-1.3 [-2.01, -0.59]	1.35 [1.09, 1.6]	1.22 [0.79, 1.66]	8.49 [4.68, 12.29]	12.29 [8.85, 15.73]
Habitat (logged)	-	-1.33 [-2.19, -0.48]	-	0.18 [-0.35, 0.71]	-	-5.71 [-9.92, -1.49]
Random effects	VC	VC	VC	VC	VC	VC
1st order	-	-	-	-	-	-
2nd order	0.451	0.458	0.375	0.367	1.060	0.253
3rd order	0.039	0.046	0.000	0.000	0.000	0.000
Block	0.446	0.034	0.000	0.000	8.147	0.000
Residuals (additive dispersion)	-	-	-	-	109.806	109.806
Fixed factors	-	0.305	-	0.007	-	7.302
$PCV_{[1st\ order]}$		-		-		-
$PCV_{[2nd\ order]}$		-1.48%		2.19%		76.11%
$PCV_{[3rd\ order]}$		-16.64%		NA		NA
$PCV_{[Block]}$		92.43%		NA		100.00%
$PCV_{[Residuals]}$		-		-		0.00%
$R^2_{GLMM(m)}$		9.64%		1.49%		6.22%
$R^2_{GLMM(c)}$		NA		NA		6.44%
AIC	543.0	540.5	780.2	781.8	824.6	819.0
BIC	562.9	560.9	790.9	795.2	838.0	835.1
Spatial autocorrelation (residuals)						
x-intercept		-0.23 [-1.63, 1.64]		-0.58 [-8.86, 8.75]		-1.36 [-9.13, 1.42]
y-intercept		-0.03 [-0.05, 0.05]		-0.13 [-0.1, 0.11]		-0.04 [-0.27, 0.11]

Supplementary Table 3 cont.

Response:	(g) Invertebrate biomass<br>Linear mixed model	
Model name	Null model	Full model
Fixed effects	b [95% CI]	b [95% CI]
Intercept	0.22 [0.14, 0.31]	0.11 [0.05, 0.18]
Habitat (logged)	-	0.18 [0.1, 0.25]
Random effects	VC	VC
1st order	-	-
2nd order	0.000	0.000
3rd order	0.000	0.000
Block	0.008	0.000
Residuals (additive dispersion)	0.055	0.055
Fixed factors	-	0.006
$PCV_{[1st\ order]}$		-
$PCV_{[2nd\ order]}$		NA
$PCV_{[3rd\ order]}$		NA
$PCV_{[Block]}$		100.00%
$PCV_{[Residuals]}$		0.63%
$R^2_{GLMM(m)}$		10.14%
$R^2_{GLMM(c)}$		10.14%
AIC	12.0	8.9
BIC	28.4	28.6
Spatial autocorrelation (residuals)		
x-intercept		-3.91 [-6.23, 1.92]
y-intercept		-0.02 [-0.11, 0.06]

For full models, the intercept represents the primary forest control. CI = Confidence interval; PCV = Proportion change in variance; NA = Not applicable/available; AIC = Akaike Information Criterion; BIC = Bayesian Information Criterion; VC = Variance components

Supplementary Table 4. Mixed effects modelling testing for the effects of logging on five aspects of the physical and biotic environment: (a) leaf area index, (b) maximum air temperature, (c) minimum relative humidity, (d) fungal hyphae abundance and (e) fruiting and flowering.

Response:	(a) Leaf Area Index<br>Linear mixed model		(b) Maximum air temperature<br>Linear mixed model		(c) Minimum relative humidity<br>Linear mixed model	
Model name	Null model	Full model	Null model	Full model	Null model	Full model
Fixed effects	*b* [95% CI]	*b* [95% CI]	*b* [95% CI]	*b* [95% CI]	*b* [95% CI]	*b* [95% CI]
Intercept	3.95 [3.77, 4.12]	4.31 [3.97, 4.65]	27.69 [27.16, 28.22]	26.56 [25.55, 27.58]	85.34 [83.69, 86.99]	89.31 [86.06, 92.55]
Habitat (logged)	-	-0.46 [-0.84, -0.08]	-	1.4 [0.28, 2.51]	-	-4.89 [-8.41, -1.36]
Random effects	VC	VC	VC	VC	VC	VC
1st order	-	-	-	-	-	-
2nd order	-	-	0.1254	0.1255	1.9180	2.2300
3rd order	0.0713	0.0577	0.8491	0.8094	9.4830	7.6170
Block	0.0150	0.0000	0.5927	0.1371	1.9440	0.0000
Residuals (additive dispersion)	0.4306	0.4300	2.2110	2.2145	53.3450	53.5600
Fixed factors	-	0.0348	-	0.0265	-	3.4647
$PCV_{[1st\ order]}$		-		-		-
$PCV_{[2nd\ order]}$		-		-0.08%		-16.27%
$PCV_{[3rd\ order]}$		19.10%		4.68%		19.68%
$PCV_{[Block]}$		100.00%		76.87%		100.00%
$PCV_{[Residuals]}$		0.13%		-0.16%		-0.40%
$R^2_{GLMM(m)}$		6.66%		7.67%		5.18%
$R^2_{GLMM(c)}$		17.70%		37.79%		19.90%
AIC	227.8	226.0	963.9	960.0	1641.2	1633.9
BIC	238.3	239.1	981.4	981.0	1658.5	1654.6
Spatial autocorrelation (residuals)						
x-intercept		-9.63 [-10.71, 4]		-1.43 [-5.46, 1.33]		-1.37 [-5.78, 3.59]
y-intercept		-0.04 [-0.17, 0.1]		-0.06 [-0.13, 0.01]		-0.03 [-0.11, 0.04]

Supplementary Table 4 cont.

Response:	(d) Fungal hyphae abundance Poisson mixed model		(e) Fruiting and flowering Binomial mixed model	
Model name	Null model	Full model	Null model	Full model
Fixed effects	b [95% CI]	b [95% CI]	b [95% CI]	b [95% CI]
Intercept	2 [1.52, 2.48]	2.8 [1.96, 3.64]	0.54 [-0.2, 1.29]	-0.83 [-2.17, 0.5]
Habitat (logged)	-	-1.03 [-1.98, -0.08]	-	1.72 [0.23, 3.22]
Random effects	VC	VC	VC	VC
1st order	-	-	-	-
2nd order	-	-	-	-
3rd order	0.3676	0.3636	0.0000	0.0000
Block	0.3994	0.2398	0.7633	0.3175
Residuals (additive dispersion)	-	-	-	-
Fixed factors	-	0.1320	-	0.3848
$PCV_{[1st\ order]}$		-		-
$PCV_{[2nd\ order]}$		-		-
$PCV_{[3rd\ order]}$		1.09%		NA
$PCV_{[Block]}$		39.96%		58.40%
$PCV_{[Residuals]}$		-		-
$R^2_{GLMM(m)}$		15.30%		9.64%
$R^2_{GLMM(c)}$		NA		17.59%
AIC	844.5	842.7	138.4	135.9
BIC	852.6	853.5	146.4	146.5
Spatial autocorrelation (residuals)				
x-intercept		-2.21 [-8.62, 1.02]		-8.46 [-9.21, 4.13]
y-intercept		-0.12 [-0.32, 0.02]		-0.01 [-0.16, 0.16]

For full models, the intercept represents the primary forest control. CI = Confidence interval; PCV = Proportion change in variance; NA = Not applicable/available; AIC = Akaike Information Criterion; BIC = Bayesian Information Criterion; VC = Variance components

Supplementary Table 5. Mixed effects modelling testing for the effects of logging on six aspects of the functional composition of invertebrate communities: (a) foraging ant abundance, (b) foraging ant species richness, (c) foraging ant body size, (d) abundance of beetle predators, (e) abundance of foraging ant specialist predators and (f) abundance of invertebrate herbivores.

Response:	(a) Foraging ant abundance Poisson mixed model (log link)		(b) Foraging ant species richness Poisson mixed model (log link)		(c) Foraging ant body size Linear mixed model	
Model name	Null model	Full model	Null model	Full model	Null model	Full model
Fixed effects	b [95% CI]	b [95% CI]	b [95% CI]	b [95% CI]	b [95% CI]	b [95% CI]
Intercept	3.99 [3.81, 4.18]	3.96 [3.44, 4.49]	1.3 [1.21, 1.4]	1.61 [1.4, 1.82]	0.21 [0.14, 0.27]	0.37 [0.22, 0.53]
Habitat (logged)	-	0.03 [-0.53, 0.6]	-	-0.34 [-0.56, -0.12]	-	-0.19 [-0.36, -0.02]
Random effects	VC	VC	VC	VC	VC	VC
1st order	-	-	-	-	-	-
2nd order	0.219	0.219	0.000	0.000	0.010	0.010
3rd order	0.000	0.000	0.000	0.000	0.000	0.000
Block	0.047	0.047	0.007	0.000	0.006	0.003
Residuals (additive dispersion)	-	-	-	-	0.038	0.038
Fixed factors	-	0.000	-	0.009	-	0.003
$PCV_{[1st\ order]}$		-		-		-
$PCV_{[2nd\ order]}$		0.00%		NA		-1.45%
$PCV_{[3rd\ order]}$		NA		NA		79.67%
$PCV_{[Block]}$		0.21%		100.00%		50.41%
$PCV_{[Residuals]}$		-		-		-0.06%
$R^2_{GLMM(m)}$		0.03%		3.72%		5.32%
$R^2_{GLMM(c)}$		NA		NA		30.30%
AIC	4230.3	4232.3	776.2	789.6	-20.9	-20.0
BIC	4243.7	4249.0	770.6	787.3	-4.2	0.0
Spatial autocorrelation (residuals)						
x-intercept		-0.96 [-4.85, -0.43]		3.5 [-4.44, 7.08]		-4.72 [-5.49, 2.04]
y-intercept		-0.11 [-0.2, -0.03]		0.02 [-0.08, 0.13]		-0.05 [-0.13, 0.04]

Supplementary Table 5 cont.

Response:	(d) Beetle predators<br>Poisson mixed model (log link)		(e) Foraging ant specialist predators<br>Poisson mixed model (log link)		(f) Invertebrate herbivores<br>Poisson mixed model (log link)	
Model name	Null model	Full model	Null model	Full model	Null model	Full model
Fixed effects	b [95% CI]	b [95% CI]	b [95% CI]	b [95% CI]	b [95% CI]	b [95% CI]
Intercept	1.49 [1.13, 1.84]	2 [1.65, 2.35]	-0.38 [-1.19, 0.43]	1.67 [-0.5, 3.85]	2.48 [2.21, 2.76]	1.81 [1.16, 2.45]
Habitat (logged)	-	-0.68 [-1.09, -0.28]	-	-2.24 [-4.54, 0.05]	-	0.78 [0.09, 1.46]
Random effects	VC	VC	VC	VC	VC	VC
1st order	-	-	-	-	-	-
2nd order	0.412	0.413	2.988	3.123	-	-
3rd order	0.023	0.015	2.657	2.488	0.242	0.230
Block	0.096	0.003	0.279	0.043	0.071	0.019
Residuals (additive dispersion)	-	-	-	-	-	-
Fixed factors	-	0.094	-	0.400	-	0.061
$PCV_{[1st\ order]}$		-		-		-
$PCV_{[2nd\ order]}$		-0.31%		-4.51%		NA
$PCV_{[3rd\ order]}$		32.90%		6.36%		0.048704792
$PCV_{[Block]}$		97.05%		84.55%		0.732465351
$PCV_{[Residuals]}$		-		-		-
$R^2_{GLMM(m)}$		12.87%		5.75%		0.1558346
$R^2_{GLMM(c)}$		NA		NA		NA
AIC	1495.8	1492.7	1330.2	1328.8	2738.8	2736.9
BIC	1508.9	1509.1	1343.5	1345.5	2746.9	2747.6
Spatial autocorrelation (residuals)						
x-intercept		-0.77 [-4.76, -0.01]		-1.17 [-4.62, -0.09]		-1.81 [-7.58, 8.45]
y-intercept		-0.08 [-0.16, 0]		-0.08 [-0.16, 0]		-0.1 [-0.1, 0.09]

For full models, the intercept represents the primary forest control. CI = Confidence interval; PCV = Proportion change in variance; NA = Not applicable/available; AIC = Akaike Information Criterion; BIC = Bayesian Information Criterion; VC = Variance components

Supplementary Table 6. Mixed effects modelling testing for the effects of logging on five aspects of the functional composition of vertebrate communities: (a) small mammal capture rate, (b) frog occupancy, (c) insectivorous bird abundance, (d) granivorous bird occupancy and (e) insectivorous bat abundance.

Response:	(a) Small mammal capture rate<br>Linear mixed model		(b) Frog occupancy<br>Binomial mixed model (logit link)		(c) Insectivorous bird abundance<br>Poisson mixed model (log link)	
Model name	Null model	Full model	Null model	Full model	Null model	Full model
Fixed effects	*b* [95% CI]	*b* [95% CI]	*b* [95% CI]	*b* [95% CI]	*b* [95% CI]	*b* [95% CI]
Intercept	0.26 [0.16, 0.35]	0.14 [0.03, 0.24]	-3.56 [-5.38, -1.73]	-34.47 [-521.26, 452.32]	2.83 [2.75, 2.91]	2.61 [2.47, 2.75]
Habitat (logged)	-	0.18 [0.06, 0.29]	-	31.84 [-454.95, 518.63]	-	0.27 [0.12, 0.42]
Random effects	VC	VC	VC	VC	VC	VC
1st order	0.0004	0.0004	-	-	-	-
2nd order	0.0014	0.0014	2.782	1.107	-	-
3rd order	0.0087	0.0094	0.000	0.000	0.0084	0.0056
Block	0.0100	0.0008	0.000	0.000	0.0068	0.0000
Residuals (additive dispersion)	0.0398	0.0398	-	-	-	-
Fixed factors	-	0.0053	-	202.749	-	0.0098
$PCV_{[1st\ order]}$		-3.76%		-		-
$PCV_{[2nd\ order]}$		-1.84%		60.21%		-
$PCV_{[3rd\ order]}$		-7.44%		100.00%		33.27%
$PCV_{[Block]}$		92.07%		100.00%		100.00%
$PCV_{[Residuals]}$		0.01%		-		-
$R^2_{GLMM(m)}$		9.30%		97.88%		13.48%
$R^2_{GLMM(c)}$		30.22%		98.41%		NA
AIC	-377.0	-375.9	106.4	100.8	668.7	660.7
BIC	-346.2	-340.0	119.6	117.2	679.7	674.4
Spatial autocorrelation (residuals)						
x-intercept		-0.54 [-4.1, 0.36]		-0.48 [-2.97, 53.5]		-0.69 [-8.43, 4.67]
y-intercept		-0.02 [-0.04, 0.01]		-0.03 [-0.11, 0.07]		-0.01 [-0.15, 0.14]

Supplementary Table 6 cont.

Response:	(d) Granivorous bird occupancy Binomial mixed model (logit link)		(e) Insectivorous bat abundance Poisson mixed model (log link)	
Model name	Null model	Full model	Null model	Full model
Fixed effects	*b* [95% CI]	*b* [95% CI]	*b* [95% CI]	*b* [95% CI]
Intercept	3.61 [2.46, 4.76]	0 [0, 0]	0.75 [0.52, 0.98]	0.98 [0.33, 1.64]
Habitat (logged)	-	0 [0, 0]	-	-0.26 [-0.96, 0.44]
Random effects	VC	VC	VC	VC
1st order	-	-	-	-
2nd order	-	-	-	-
3rd order	0.0000	0.0000	0.2431	0.2427
Block	0.0000	0.0000	0.0000	0.0000
Residuals (additive dispersion)	-	-	-	-
Fixed factors	-	-	-	0.0076
$PCV_{[1st\ order]}$		-		-
$PCV_{[2nd\ order]}$		-		-
$PCV_{[3rd\ order]}$		100.00%		0.16%
$PCV_{[Block]}$		NA		NA
$PCV_{[Residuals]}$		-		-
$R^2_{GLMM(m)}$		97.68%		1.19%
$R^2_{GLMM(c)}$		97.68%		NA
AIC	33.7	0.0	419.5	421.0
BIC	42.0	0.0	427.1	431.2
Spatial autocorrelation (residuals)				
x-intercept		0 [0, 0]		-2.28 [-9.42, 5.5]
y-intercept		0 [0, 0]		-0.11 [-0.21, 0.07]

For full models, the intercept represents the primary forest control. CI = Confidence interval; PCV = Proportion change in variance; NA = Not applicable/available; AIC = Akaike Information Criterion; BIC = Bayesian Information Criterion; VC = Variance components

Supplementary Reference

1 Ewers, R. M. *et al.* A large-scale forest fragmentation experiment: the Stability of Altered Forest Ecosystems Project. *Philos. Trans. R. Soc. B-Biol. Sci.* **366**, 3292-3302, (2011).
